# Intraindividual variability (IIV) in an animal model of ADHD - the Spontaneously Hypertensive Rat

**DOI:** 10.1186/1744-9081-6-56

**Published:** 2010-10-06

**Authors:** Guy ML Perry, Terje Sagvolden, Stephen V Faraone

**Affiliations:** 1 Department of Medicine, SUNY Upstate Medical University, 750 E Adams St, Syracuse, NY 13210, USA; 2 Department of Physiology, Institute of Basic Medical Sciences, University of Oslo, NO-0317 Oslo, Norway; 3 Department of Psychiatry, SUNY Upstate Medical University, 750 E Adams St, Syracuse, NY 13210, USA; 4 Department of Medicine Neuroscience and Physiology, SUNY Upstate Medical University, 750 E Adams St, Syracuse, NY 13210, USA

## Abstract

Attention-deficit/hyperactivity disorder (ADHD) is characterized by numerous behaviors including inattention, hyperactivity and impulsiveness. ADHD-affected individuals also have high intra-individual variability (IIV) in reaction time. The genetic control of IIV is not well understood. The single study of the genetics of this phenomenon in humans detected only marginal associations between genotypes at two candidate genes for ADHD and variability in response time. The Spontaneously Hypertensive Rat (SHR/NCrl) is an animal model of ADHD, expressing high activity, inattention and impulsive behavior during operant and task tests. The SHR might be useful for identifying genes for variability, but it is not known whether it also expresses high IIV, as is symptomatic of ADHD. We therefore conducted an investigation of IIV in the SHR. We used 16 SHR/NCrl rats and 15 Wistar-Kyoto (WKY/Nico) controls applying a reinforcement schedule used in the validation of the SHR as an animal model of ADHD. We represented IIV as the average absolute deviation of individual behavior within the five 18-min segments of each experimental session from the average behavioral trait value within that session ('individual phenotypic dispersion', *PD_i_*). *PD_i _*for hyperactivity, impulsiveness and inattention in the SHR and WKY rats was analyzed using nonparametric ranking by experimental session. SHR/NCrl rats had higher *PD_i _*than WKY/Nico controls for impulsiveness and inattention. There was a significant upward trend for *PD_i _*over experimental segments within sessions for attention in SHR rats, but not in WKY. *PD_i _*for hyperactivity was correlated with *PD_i _*for impulsiveness and we therefore excluded observations associated with short IRTs (< 0.67s); dispersion in hyperactivity outside this interval was also significantly higher in SHR rats than in WKY rats. Some studies indicate the sharing of symptoms of hyperactivity and impulsiveness in SHR and ADHD-affected humans; high IIV in operant behavioral metrics suggests that the SHR may be useful in elucidating the genetic basis for IIV in humans.

## Findings

Attention-Deficit/Hyperactivity Disorder (ADHD) is a common, highly heritable [1] and costly ($US 67B-116B) [1,2] disorder characterized by hyperactivity, impulsiveness and inattention. ADHD is associated with neuropsychological dysfunction [1], structural [3] and functional [4] brain anomalies. In addition to deficits in neuropsychological and psychosocial functions, patients with ADHD have greater variability in task reaction time [5], spatial placement challenges [6] performance tasks and Go-NoGo tasks [7] compared to unaffected individuals. Since this is measured as variability in individual subjects within the task, it is termed intra-individual variability (IIV). Although initially considered a form of residual experimental or measurement error, IIV is frequently observed and may be an endophenotype of ADHD [8-10]. Little is known of the control of this phenomenon, but there is some evidence that it may be genetic. Cho et al [11] found marginally higher variability in response time during continuous performance tests for CC and GG genotypes at the DraI and MspI polymorphisms, respectively, in the alpha-2A-adrenergic receptor. Individuals inheriting the Val allele at catechol-O-methyltransferase had higher variability during executive functioning tests [12].

Many studies have shown that the Spontaneously Hypertensive Rat (SHR/NCrl) shows the full range of ADHD-like symptoms, including increased motor activity, impulsiveness (short inter-response time) and decreased attention [13-17]. It also shows biological features that parallel those seen in ADHD patients such as smaller brains [18] and altered activity in dopaminergic, norepinephrine and ionic/energetic exchange genes [15,17,19-21]. Compared to appropriate controls, SHR as well as ADHD-affected children show increased responding during a fixed-interval schedule of reinforcement as well as during extinction of learned behavior [15,16]. Because it is not known whether the SHR also exhibits variability in hyperactive, attentive and impulsive behavior, our objective was to determine whether the SHR is also a valid model of this feature of ADHD.

We compared IIV in elements of operant behavior representing activity, attention and impulsiveness in 16 SHR (Charles River, Italy; SHR/CrlNico) and 15 WKY rats (Charles River, France; WKY/Nico) in a simultaneous visual discrimination task using sixteen Campden Instruments operant chambers (see [see [22,23]]. Five-week old experimentally naïve rats were acclimatized for eight days in individual housing *with ad libitum *access to food and water before being placed in the experimental chamber for one hour for initial habituation. After the first habituation session, rats were deprived of water for 21 hours each day before each succeeding session. This is a moderate but sufficient motivational deprivation approved by the Norwegian Animal Research Authority (NARA), in accordance with Norwegian laws and regulations on live animal experimentation.

Rats were then trained to use retractable levers in the operant chambers. The behavioral procedure is described elsewhere [13,24]. In brief, the chamber had two levers. A 2.8-W cue light was located above each lever. The reinforcer (0.01 ml tap water) was delivered by a liquid dipper in a small recessed cubicle. A 2.8-W cue light was lit in the cubicle when the reinforcer was present. Opening the door into the cubicle activated a micro-switch.

A computer system (SPIDER, Paul Fray Ltd., UK) recorded behavior and scheduled reinforcers (water droplets). Reinforcers for correct responses were delivered on an unpredictable basis, at a mean of 180 s (a variable interval 180 s schedule). An extinction schedule (unassociated with any cue light) was present on the wrong lever. Each 90-min session was divided into five 18-min segments. For each segment, total number of presses on correct and incorrect levers, total number of correct (with water present) and incorrect (no water present) openings of the door into the recessed cubicle, number of reinforcers delivered, and the time between consecutive correct lever presses (inter-response time, IRT) were recorded.

We used the total number of lever presses to represent general activity (hyperactivity) and the number of responses with short IRTs (< 0.67 sec) to represent impulsiveness (see [22,23]). Attention was defined in this context as the total number of incorrect openings of the door into the recessed cubicle. These symptoms are highly similar to behavior in human ADHD; both the SHR and ADHD-affected children express short bursts of activity with short IRTs between responses [14,25] with impulsiveness increasing with task repetition [26] and general hyperactivity [25]. Responses to operant behavioral tasks are highly similar between the SHR and ADHD-affected children on the same operant schedule [14,15,25]. We therefore considered the operant tasks in this work as representative of behavior in human ADHD.

**Figure 1:**
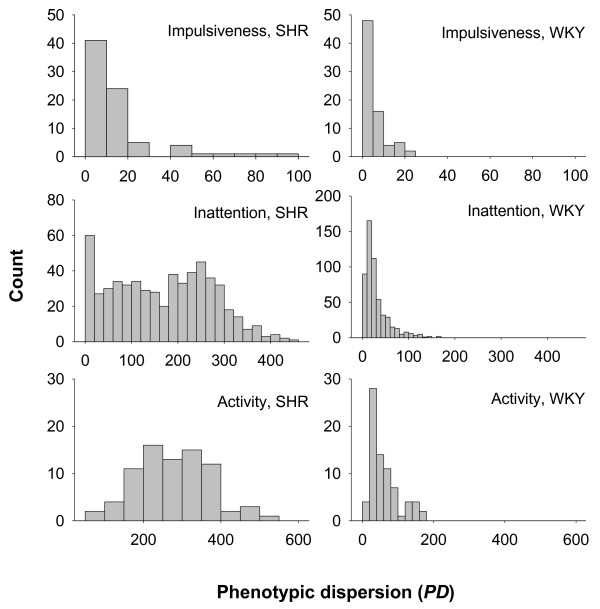
**Histogram distributions of impulsive, inattentive and hyperactive behavior in 16 Spontaneously Hypertensive Rats (SHR) and 15 control Wistar-Kyoto (WKY) rats**.

Intra-individual variability (IIV) was measured as the average absolute deviation from mean individual operant behavior by session for each trait. Individual phenotypic dispersion (*PD_i_*) was calculated as the average of absolute differences between behavior in each segment within session and the average behavior for the entire session as $PD_{i}=avg|(X_{i}^{j=1}-{\bar{X}}_{j}^{i=1})|$, where $X_{i}^{j=1}$ is the operant behavior for rat *i *within segment *j *(from 1-5) and ${\bar{X}}_{j}^{i=1}$ is the average behavior for the rat within the complete session. The distribution of *PD_i _*was strongly non-normal for all traits (Figure 1). Therefore, in order to avoid complications arising from violations of the normal distribution, we tested for differences in IIV between strains using Kruskal-Wallis nonparametric ranking of average *PD_i _*across all segments within each session, and general linear modeling [27] on average log-transformed *PD_i_*, so that one *PD_i _*value was available per individual per session. Behavioral means for each strain were estimated using general linear modeling of log-transformed *PD_i_*. Variance proportions for *PD_i _*were estimated from log-transformed averages across all sessions and segments [27]. To avoid confounding dispersion estimates for impulsiveness and hyperactivity by the inclusion of short IRTs in estimates of total activity, we excluded all activity measurements with IRT < 0.67 s in the estimation of *PD_i _*for hyperactivity.

Using repeated ANOVA on log-transformed *PD_i_*, we also tested for temporal changes in IIV by strain i) over sessions [27] for *PD_i_*, fitting effects for strain, consecutive session day (1-5) and their interaction (the 'among sessions' test), ii) by increasing segment (1-5) within session, with *PD_i _*averaged for each six-minute segment across all sessions [27] (the 'within sessions' test). Since only one session occurred per day, test i) corresponded to changes over days and test ii) to temporal changes within day.

Behavioral dispersion was different in the SHR to the WKY for all three behavioral traits (Figure 1). The SHR strain had statistically higher *PD_i _*for impulsiveness (*p *< 0.001), hyperactivity (*p *< 0.0001) and inattention (*p *< 0.0001) than WKY rats (Table 1). The proportion of variance explained by strain varied from a quarter of all variance (impulsiveness) to 60% of total variance (inattention) (Table 1).

**Table 1:** Intra-individual variability (IIV) for impulsiveness and inattention, measured as phenotypic dispersion (*PD_i_*) averaged over experimental session repeated by six-minute experimental segment in 16 Spontaneously Hypertensive Rats (SHR) and 15 Wistar-Kyoto (WKY) rats [27].

Trait	*χ*^2^	*P*	*F*	*P*	*μ*_SHR _± 95%CI	*μ*_WKY _± 95%CI
Impulsiveness	15.3	< 0.0001	15.8	0.0001	1.26 ± 0.201	0.680 ± 0.212
Hyperactivity	52.8	< 0.0001	77.6	< 0.0001	3.65 ± 0.134	2.80 ± 0.136
Inattention	111.5	< 0.0001	452.4	< 0.0001	4.77 ± 0.138	2.62 ± 0.142

Differences in dispersion were estimated using Kruskal-Wallis nonparametric ranking (*χ*^2^) and log-transformed *PD_i _*(*F*); differences in means for dispersion were estimated from log-transformed *PD_i_*. Variance was estimated from behavioral averages for individuals across all sessions and experimental segments [27].

There was no evidence that IIV increased or decreased with sessions (days) (*p *> 0.20) or that there were differences in IIV over sessions by strain (model i) (*p *> 0.40). IIV changed with time within sessions (model ii): *PD_i _*for hyperactivity was negatively correlated with the sequence of sessions over time in both strains (*p *< 0.0001; *β *= -1.17 ± 1.07). *PD_i _*for attention was strongly affected by segment within sessions (*p *< 0.0001). *PD_i _*in the SHR increased with segment (*β *= 2.40 ± 0.055) but decreased significantly in the WKY (*β *= -1.61 ± 0.057).

Our finding that *PD_i _*differed for inattention, activity and impulsiveness between the SHR and WKY suggests a genetic component to IIV. In ADHD-affected individuals, increased IIV is seen in behavior maintained by reinforcers [6,26], tests of executive functioning [12], and continuous performance and response time functioning [5,7], the latter being related to attention.

High behavioral IIV in both the SHR and ADHD-affected children also obliquely supports the validity of the SHR as a model of ADHD [8,17,19,22,28]. Intra-individual variability in ADHD has clinical implications: IIV might require restructuring of concurrent speed and accuracy demands in cognitive tasks or the division of long intervals of repetitive testing into shorter segments with more immediate reinforcement [8,11,29]. Higher *PD_i _*in the SHR relative to the WKY highly resembles dispersion for this strain in a related article [30], suggesting that differences in dispersion between these strains are consistent. Increasing dispersion in the SHR within experimental sessions suggests that the SHR's behavior becomes increasingly erratic with ongoing repetitive operant testing. Similar increases in variance over segments within experimental sessions were observed in ADHD-affected children challenged with spatial response placements at low reinforcement frequency [31].

There are several physiological pathways that might explain IIV: Castellanos [5] suggested that IIV resulted from poor regulation of neural periodicity. Russell [29] proposed that short-term IIV was due to insufficient lactate supply to highly active neurons and/or slow recovery of neuronal ionic balance, and that long-term IIV would result from poor myelination of long neurons due to lactate deficiency during development [29]. Other work suggests that IIV could result from poor joint dopaminergic/catecholaminergic regulation in the prefrontal cortex. Poor control of noradrenergic output might cause irregular adrenergic activity in the prefrontal cortex, resulting in increased noise in neuron function and increasing attention to irrelevant stimuli [11,32]. Sagvolden et al [8] proposed the 'dynamic developmental theory of ADHD', in which reduced dopamine function changes fundamental behavioral mechanisms via deficient reinforcement of successful behavior combined with deficient extinction of unsuccessful behavior. Such mechanisms would slow the association ("chunking") of simple response units into elaborate, higher-order adaptive chains [6] in which one response unit reliably precedes the next. Deficient or slowed chunking might make such patterns unreliable, resulting in intra-individual variability [10]. At this point, the basis for IIV in the SHR is not known, although these results are compatible with underlying deficiencies in factors associated with reinforcement, as in the shorter delay gradient in the SHR [33]; SHR/NCrl do also have altered activity in a number of genes involved in synaptic plasticity and learning [17,20,28]. We cannot presently discern between any of the above explanations, although behavioral variability in the SHR is strong evidence of its face validity as a model of ADHD.

## List of Abbreviations

ADHD: Attention-Deficit Hyperactivity Disorder; ANOVA: Analysis of Variance; IIV: Intra-Individual Variability; IRT: Inter-Response Time; PD_i _= Individual Phenotypic Dispersion; SHR: Spontaneously Hypertensive Rat (SHR/NCrl); WKY: Wistar-Kyoto rat (WKY/Nico)

## Competing interests

Dr. Faraone has in the past year received consulting fees and served on Advisory Boards for Eli Lilly, Ortho-McNeil and Shire Development, and has received research support from Shire and the National Institutes of Health. In previous years, Dr. Faraone has received consulting fees or has been on Advisory Boards or has been a speaker for Shire, McNeil, Janssen, Novartis, Pfizer, Ortho-McNeil and Eli Lilly. In previous years he has received research support from Eli Lilly, Shire, Pfizer and the National Institutes of Health. TS has received consulting fees or research support or has been on Advisory Boards or has been a speaker for: Shire, Janssen, and Eli Lilly. Dr Perry has no competing interests.

## Authors' contributions

GP provided the theoretical basis for this work, carried out the statistical analysis and wrote the article. TS provided the experimental data and participated in writing and editing the manuscript. SF supervised editing and the medical basis of the written work and the analysis. All authors read and approved the final manuscript.
